# Is the Membrane Lipid Matrix a Key Target for Action of Pharmacologically Active Plant Saponins?

**DOI:** 10.3390/ijms22063167

**Published:** 2021-03-20

**Authors:** Svetlana S. Efimova, Olga S. Ostroumova

**Affiliations:** Institute of Cytology of Russian Academy of Sciences, 199034 Saint Petersburg, Russia; ostroumova@incras.ru

**Keywords:** saponins, terpenes, lipid bilayers, liposomes, membrane boundary potential, lipid melting, calcein release, ion channel, gramicidin A, syringomycin E, nystatin

## Abstract

This study was focused on the molecular mechanisms of action of saponins and related compounds (sapogenins and alkaloids) on model lipid membranes. Steroids and triterpenes were tested. A systematic analysis of the effects of these chemicals on the physicochemical properties of the lipid bilayers and on the formation and functionality of the reconstituted ion channels induced by antimicrobial agents was performed. It was found that digitonin, tribulosin, and dioscin substantially reduced the boundary potential of the phosphatidylcholine membranes. We concluded that saponins might affect the membrane boundary potential by restructuring the membrane hydration layer. Moreover, an increase in the conductance and lifetime of gramicidin A channels in the presence of tribulosin was due to an alteration in the membrane dipole potential. Differential scanning microcalorimetry data indicated the key role of the sapogenin core structure (steroid or triterpenic) in affecting lipid melting and disordering. We showed that an alteration in pore forming activity of syringomycin E by dioscin might be due to amendments in the lipid packing. We also found that the ability of saponins to disengage the fluorescent marker calcein from lipid vesicles might be also determined by their ability to induce a positive curvature stress.

## 1. Introduction

Saponins have a wide spectrum of pharmacological action, including anti-inflammatory, anti-nociceptive, anti-pyretic, anti-allergic, and anti-cancer properties [1,2]. Pronounced hemolytic activity is explained by the interaction of saponins with cholesterol (CHOL) in the erythrocyte membranes [3,4]. The structural diversity of saponins determines the variety of their physico-chemical, pharmacological, and biological properties, and their application in the food, cosmetics, and pharmaceutical industries [5].

Since cell membranes are quite complex systems, model systems mimicking the structure and composition of target cell membranes have been widely used to investigate the activity of small molecules of different chemical origin, including saponins, in recent decades. The consequences of interaction of saponin and related compounds with lipid bilayers, namely, their effects on phospholipid and CHOLenriched bilayers, membrane permeability, lipid dynamics, domain formation, and disruption are summarized in [6]. 

Using differential scanning microcalorimetry of liposomes formed from pure dimyristoylphosphatidylcholine (DMPC), dimyristoylphosphatidylethanolamine, and dimyristoylphosphatidylserine, it was shown that the solasodine, diosgenin, and solanine reduce the main phase transition temperature and the cooperativity of the lipid phase transition in a concentration-dependent manner [7]. Nishikawa et al. [8] showed a digitonin-induced decrease in the order parameter of spin-labeled phosphatidylcholine and the restoration of the phase transition of phosphatidylcholine in the presence of CHOL in lipid vesicles. A controversiality in order parameters of lipids in the presence of saponins measured by EPR, fluorescence spectroscopy, and ^2^H-NMR was explained by Lorent et al. [6] taking into account the different acquisition time scales of these methods and a dependence of lipid motions on the CHOL content.

It was shown that escin, α-hederin, and its sapogenin hederagenin at a concentration up to 5 mol% can induce the formation of visible lipid ordered domains in a pure DMPC or lipid mixture of DMPC and CHOL (3:1) [9,10]. By atomic force microscopy and surface plasmon resonance measurements Naruse et al. [11] revealed that a steroid saponin from the starfish *Asterias amurensis*, named as cofactor for acrosome reaction-inducing substance (Co-ARIS), is colocalized with ganglioside clusters.

Three principal mechanisms involved in the saponin-induced membrane permeabilization were postulated by Lorent et al. [6]: 1) membrane rearrangement induced by the formation of a new lipid phase enriched with saponins and sterols; 2) toroidal pores formed by saponin and CHOL aggregates; 3) membrane permeabilization due to induction of the positive curvature stress. The first mechanism was proposed for several glycoalkaloids from potato and tomato [12]. The dependence of cation/anion selectivity of avicin channels on membrane lipid composition [13] might imply the formation of saponin/phospholipid toroidal pores. The processes of formation of transmembrane pores in CHOL-containing phosphatidylcholine membranes by α-hederin and distortion of lipid rafts enriched with sphingolipids and CHOL by dioscin might be described in terms of the third mechanism [9,14,15]. The pores induced by digitonin and commercial Merck saponin in diphytanoylphosphatidylcholine bilayers with and without CHOL might also suggest the induction of micellar structures [16]. However, the threshold concentration leading to the appearance of saponin-induced current fluctuations in sterol-enriched bilayers was three orders lower than in membranes without CHOL indicating that these saponins preferentially interact with more ordered CHOL-enriched regions. 

The aim of this work was to determine the mechanisms of interaction of various saponins and related compounds (sapogenins and alkaloids) with model lipid membranes. To elucidate the role of the lipophilic core of the saponin molecules, we tested steroid and triterpenic sapogenins, as well as a steroid alkaloid. To understand the importance of the hydrophilic sugar part, we studied the saponins containing various numbers of saccharide moieties, as well as sapogenins. We used phosphatidylcholine, plasmanylcholine, phosphatidylglycerol, and CHOL-enriched bilayers. To achieve the goal, we revealed the maximum threshold concentrations of the tested compounds required to increase the ion permeability of model lipid membranes, the chemical-induced changes in the bilayer boundary potential, the kinetics and concentration dependence of the leakage of the fluorescent dye calcein from vesicles in the presence of saponins and related compounds, their effects on the lipid thermotropic behavior, and consequently, the relationship between the molecule structure and changes in the physical properties of the membranes. In addition, we studied the physicochemical characteristics of model lipid membranes altered by saponins that play a decisive role in affecting the formation and functionality of reconstituted ion channels formed by antimicrobial agents.

For the first time, the ability of saponins to influence the transmembrane distribution of electrical potential and, thereby, regulate the ion transport by voltage-sensitive channels was demonstrated. It was also found, for the first time, that changes in the elastic properties of the lipid matrix in the presence of saponins (in particular, dioscin) might determine the effects on the mechanosensitive ion channels. The pore-forming activity of a number of saponins and related compounds was established. It was assumed that their ability to increase the permeability of membranes to ions and large organic molecules was due to the lipid disordering and the induction of membrane curvature stress and the formation of micellar-like structures. The relationships between molecule structure and the agent efficacy to affect a variety of physical characteristics of the lipid bilayer were established.

## 2. Results and Discussion

### 2.1. The Effect of Saponins and Related Compounds on the Ion Permeability of Lipid Bilayers

**Figure 1:**
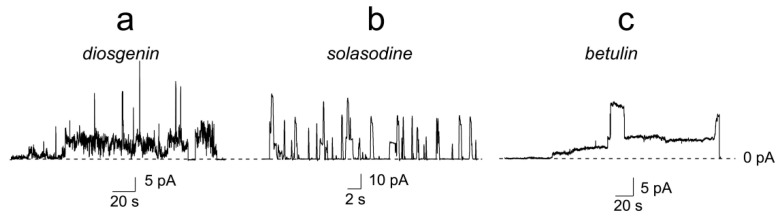
Current fluctuations corresponding to the openings and closures of single ion-permeable pores induced by 150 μM of diosgenin (**a**), solasodine (**b**), and betulin (**c**). Lipid bilayers were formed from DPhPC and bathed in 0.1 M KCl (pH 7.4). *V* = 100 mV.

The ability of saponins and related compounds to increase the ion permeability of planar lipid bilayers was studied. An addition of the compounds to the aqueous solution at the *cis*-side of the DPhPC-membranes, bathed in 0.1 M KCl (pH 7.4), up to 150–200 µM led to bilayer disruption (Table 1). Diosgenin, solasodine, and betulin demonstrated an ability to induce step-like current fluctuations that should be attributed to the formation of ion-permeable pores before the membrane disintegration (Figure 1). The conductance of the pores varied in the range from 20 to 400 pS at a transmembrane voltage of 100 mV. 

An introduction of the CHOL to the DPhPC membranes led to a decrease in the agent threshold concentrations, causing the bilayer to rupture at 75–150 µM (Table 1). The decrease in the threshold concentrations of the chemicals might be due to their enhanced interaction with CHOL-enriched lipid bilayers compared to sterol-free membranes. Moreover, all steroids (digitonin, tribulosin, dioscin, diosgenin, and solasodine) and only one triterpene betulin demonstrated the ability to form ion permeable pores in DPhPC/CHOL-bilayers (Figure S1 Supplementary materials). The amplitude of the observed step-like current fluctuations varied in the range from 50 to 500 pS at a transmembrane voltage of 100 mV. At the same time, other triterpenes, escin, uvaol, and lupeol, did not show pore forming activity in either pure DPhPC or CHOL-enriched bilayers. The induction of the micellar-like structures in the presence of saponins (in particular, digitonin) in both CHOL-free and CHOL-enriched bilayers that resulted in the pore formation was proposed by Bangham et al. [17] and Gogelein and Huby [16]. 

### 2.2. The Influence of Saponins and Related Compounds on the Boundary Potential of Model Lipid Membranes

**Figure 2:**
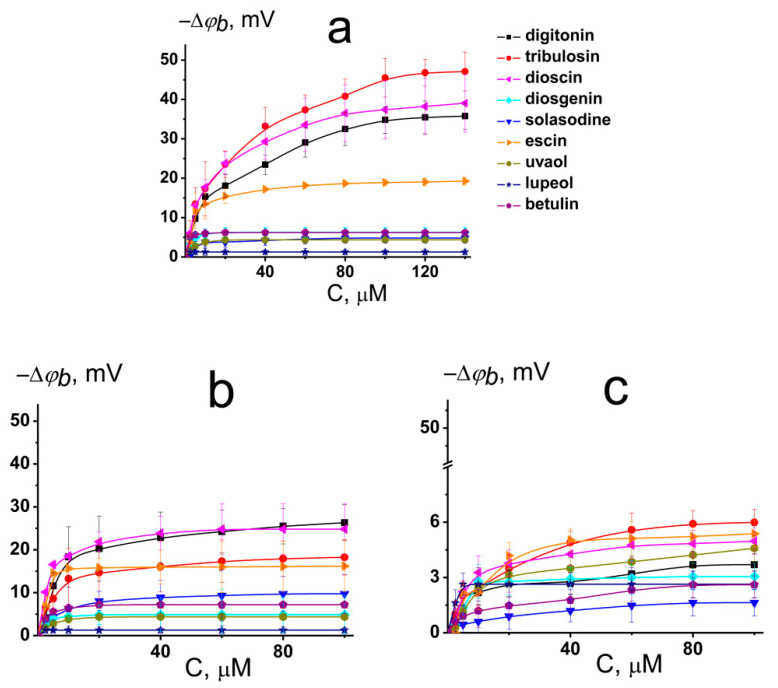
Dependence of the decrease in the boundary potential of the membrane (−Δφ*_b_*) on the concentration (*C*) of different saponins and related compounds in the membrane bathing solution. The membranes were made from DPhPC (**a**), DPhPC/CHOL (**b**) and HOPC (**c**), bathed in 0.1 M KCl (pH 7.4), and modified by nonactin. *V* was equal to 50 mV. The relation between the color symbol and the compound is given on the figure.

Figure 2a illustrates the dependence of changes in the boundary potential of DPhPC bilayers on the concentration of the tested compounds. The curves presented in Figure 2a are close to linear at low concentrations and tend toward saturation at high concentrations. A Langmuir adsorption isotherm was used to describe the adsorption of the saponins and related compounds to lipid bilayers as a first-order approximation. Table 1 and Table S1 (Supplementary materials) show the characteristic parameters of the Langmuir adsorption isotherm, namely, the maximum change in the φ*_b_* at an infinitely high concentration of the agent (−Δ*φ_b_*(max)) and its desorption constant that provides a meaningful measure of the affinity of the tested compound toward the lipid phase.

We found that under physiological conditions (0.1 M KCl, pH 7.4) digitonin, tribulosin, and dioscin caused a significant decrease in the boundary potential of DPhPC membranes. The corresponding absolute values of the maximum decrease in the boundary potential were equal to 36, 47, and 39 mV, respectively (Figure 2a, Table 1). The addition of escin to the membrane-bathing solutions led to an approximately 20 mV decrease in the boundary potential of DPhPC bilayers. It was found that all tested sapogenins (diosgenin, solasodine, uvaol, lupeol, and betulin) did not affect the electrical properties of model lipid membranes (the maximum change in the boundary potential did not exceed 6 mV) (Figure 2a, Table 1). Obtained *K*-values are in the range from 6 to 17 μM (Table S1 Supplementary material) indicating that saponins can reduce the boundary potential of phosphatidylcholine bilayers at the concentrations higher than plant flavonoids and lower than some alkaloids [18,19,20,21]. The dissociation constant *K* calculated from the *φ_b_*-changes describes only the electrical aspects of adsorption of the saponins to lipid membranes and does not take into account the adsorption, which does not lead to a change in the transmembrane distribution of the electrical potential.

The membrane boundary potential is the sum of the surface and dipole components [22,23]. The first one is attributed to the electrical charges of membrane forming molecules and the shielding of membrane surface charge by ions in the surrounding media. The last one is a manifestation of a nonrandom orientation of the lipid and water dipoles on the interphase. At pH 7.4, digitonin and dioscin present in aqueous solution only in neutral form (the electrical charge of the molecules predicted by ChemAxon is equal to 0) (Table 1). This means that changes in the membrane boundary potentials upon addition of the saponins were mainly due to changes in the bilayer dipole potential. The logarithms of the agent octanol/water distribution coefficients at pH 7.4 (LogD_o/w_) predicted by the ChemAxon calculation are presented in Table 1. No correlation between the values of LogD_o/w_ and −Δφ_b_(max) of the DPhPC-bilayer was found, which indicates that the dipole-modifying action of saponins is not related to their lipophilicity.

The introduction of CHOL into membrane composition was accompanied by a 25–50% decrease in the dipole-modifying ability of digitonin, tribulosin, dioscin, and escin (Figure 2b, Table 1). This may be due to the higher rigidity of CHOL-enriched bilayers or a difference in hydration water dynamics in CHOL-free and CHOL-enriched lipid membranes [24].

The replacement of phosphatidylcholine for its ether analog, HOPC, led to the abolishment of the modifying activity of the saponins (Figure 2c). The changes in φ*_b_* of HOPC-bilayers due to digitonin, tribulosin, solasodine, diosgenin, dioscin, escin, uvaol, lupeol, and betulin adsorption did not exceed 6 mV (Table 1). The membranes made from ether lipids are characterized by a lower dipole potential than membranes that are formed from ester lipids [22]. The explanation for the discrepancy might be related to a difference in the structure of the DPhPC and HOPC hydration layers [25,26]. Probably, saponins, digitonin, tribulosin, dioscin, and escin affect the dipole potential of the membrane by altering the membrane hydration layer due to their hydrophilic sugar parts.

### 2.3. The Effects of Saponins and Related Compounds on the Voltage-Sensitive ion Channels Formed by Antimicrobial Peptide

Additionally, we tested an idea that saponins affect ion channels via alterations in the electrical properties of membranes. To verify this possibility, we used ion channels formed by an antimicrobial peptide, gramicidin A (GrA), which is known to be sensitive to alterations in the transmembrane distribution of electrical potentials. According to the literature data [21,27], flavonoid phloretin and alkaloid dihydrocapsaicin, known to decrease the membrane dipole potential, led to an increase in the amplitude and lifetime of cation selective GrA channels.

**Figure 3:**
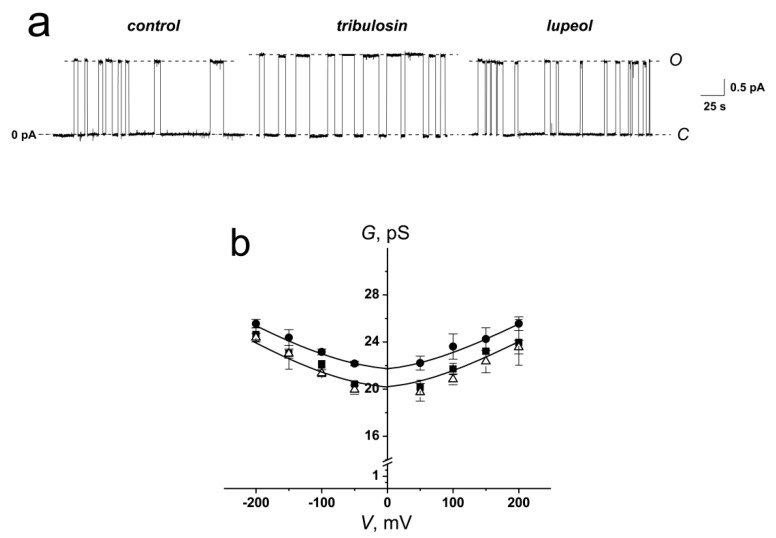
(**a**) Current fluctuations corresponding to the openings and closures of single GrA channels in the absence (*control*) and presence of 50 μM tribulosin or lupeol. *V* was equal to 100 mV. *C*—closed state of the channel, *O*—open state of the channel. (**b**) *G*(*V*) curves of single GrA channels in the absence (■) and presence of 50 μM tribulosin (●) and lupeol (Δ). The membranes were composed of DPhPC and bathed in 1.0 M KCl (pH 7.4).

Figure 3a demonstrates the effects of tribulosin and lupeol on the GrA channels reconstituted into DPhPC-bilayers bathed in 1.0 M KCl at pH 7.4. The high ionic strength of the solution corresponds to the conditions for substantial screening of the surface charge of the membrane, which allows to study the effects of changing the membrane dipole potential alone. One can see that tribulosin increased the channel amplitude and lifetime, while the lupeol did not affect the pore conductance. Figure 3b presents the conductance-voltage curves of GrA channels in the absence (control) and in the presence of tribulosin and lupeol. One can see that modifiers practically did not affect the shape of the *G*-*V* curve of single channels. The magnitudes of the conductance of single GrA channels at 100 mV in the presence and absence of tribulosin and lupeol are presented in Table 2. One can notice that the addition of tribulosin to the DPhPC-membrane bathing solution led to a 10% increase in GrA channel conductance, while lupeol did not change it. The GrA lifetime is 1.5-fold higher in the presence of tribulosin than in the absence of any modifiers and in the presence of lupeol (Table 2). Thus, changes in bilayer electrostatics under the action of tribulosin might underlie the increase in the conductance and lifetime of GrA pores. The absence of lupeol effects on the GrA pores should be related to the absence of dipole-modifying properties of the sapogenin.

### 2.4. The Effects of Saponins and Related Compounds on Lipid Melting

**Figure 4:**
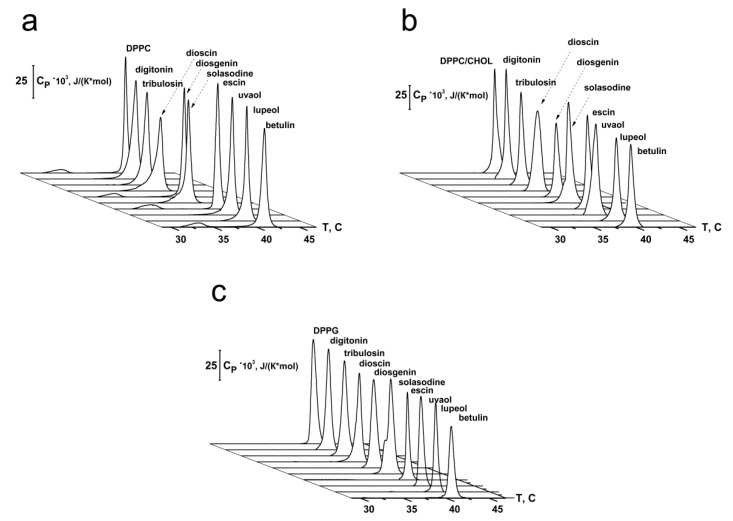
Heating thermograms of DPPC (**a**), DPPC/CHOL, (**b**) and DPPG (**c**) unilamellar liposomes in the absence and presence of saponins and related compounds at the concentration of 50 μM.

Differential scanning microcalorimetry of unilamellar lipid vesicles was made to test the effects of saponins and related compounds on the packing density of membrane-forming lipids. Figure 4 shows typical heating thermograms of giant unilamellar vesicles composed of DPPC in the absence and presence of 50 μM of tested compounds. In the absence of any agents, the temperatures of the pre-transition to ripple phase and the main phase transition of DPPC were equal to 33.9 and 41.2 °C (*T_m_*), respectively. The half-width of the peak was about 0.6 °C (*T_1/2_*), and the enthalpy of the main phase transition was about 9 kcal/mol (Δ*H*) (Figure 4a). The main characteristics of the endotherm of DPPC melting are in good agreement with the literature data [28]. The addition of digitonin, tribulosin, solasodine, dioscin, uvaol, and lupeol led to the abolishment of the pre-transition of DPPC, while in the presence of diosgenin, escin, and betulin the peak corresponding to DPPC pretransition from gel to ripple phase was observed (Figure 4a). Addition of digitonin, tribulosin, dioscin, diosgenin, and solasodine caused a significant decrease in *T_m_* of 0.4–0.9 °C and a marked increase in the *T_1/2_* of 0.3–0.7 °C (Table 3). Uvaol, lupeol, and betulin demonstrated less influence on the thermotropic behavior of DPPC: the *T_m_* was decreased by 0.2–0.3 °C and *T_1/2_* was increased by 0.1–0.2 °C (Table 3). Escin did not change the main characteristics of the DPPC melting endotherm. The enthalpy of the main phase transition of DPPC in the presence of the tested compounds was not practically changed (-∆∆*H* ranged from 0.1–0.6 kcal/mol) (Table S2 Supplementary materials). Thus, differential scanning microcalorimetry data indicated that steroid saponins (digitonin, tribulosin, dioscin), steroid sapogenin diosgenin, and steroid alkaloid solasodine were more effective in DPPC-membranes than triterpenic saponin escin, and triterpenic sapogenins (uvaol, lupeol, and betulin). The dependence of the effects on the structure of the sapogenin (steroid or triterpenic) may indicate that saponins and related compounds might affect lipid melting through immersion into the membrane and disordering of the lipids. The abolishment of the pre-transition of highly hydroxylated DPPC upon the addition of some agents indicate that tested compounds might also influence the lipid phase behavior by restructuring the membrane hydration layer.

Further, the effect of saponins and related compounds on the temperature dependence of the phase transition of a binary mixture of DPPC and CHOL (90/10 mol%) was investigated (Figure 4b). The temperature of the main phase transition of the mixture was about 40.0 °C with a half-width of the peak of about 0.7 °C, and the enthalpy was about 9.5 kcal/mol (Figure 4b). Digitonin, tribulosin, dioscin, diosgenin, and solasodine were characterized by less significant effects on the thermotropic behavior of the DPPC/CHOL-mixture compared to pure DPPC: the *T_m_* of DPPC/CHOL decreased by 0.2–0.5 °C and *T_1/2_* increased by 0.1–0.5 °C (Table 3). The data obtained with digitonin agree with the results by Nishikawa et al. [8], who showed the decrease in the order parameter of spin-labeled phosphatidylcholine and restoration of the phase transition of phosphatidylcholine in CHOL-enriched liposomes in the presence of the saponin. In contrast, uvaol, lupeol, and betulin demonstrated more pronounced effects on the DPPC/CHOL mixture than on pure DPPC: the *T_m_* decreased by 0.4–0.8 °C and *T_1/2_* increased by 0.1–0.3 °C (Table 3). Thus, steroids were characterized by less pronounced effects in CHOL-enriched bilayers than in CHOL-free membranes, while triterpenes showed an inverse relationship. Triterpenic saponin escin was ineffective toward the thermotropic behavior of lipids independent of the sterol presence; it did not alter the main characteristics of the DPPC/CHOL melting endotherm similar to pure DPPC (Figure 4b, Table 3). All tested compounds weakly decreased the enthalpy of the main phase transition of DPPC/CHOL (Table S2 Supplementary materials) (decrease in ∆∆*H* was equal to 0.5–1.5 kcal/mol). More probably, this demonstrates the ability of the agents to sequestrate a small amount of sterol from the lipid bilayer.

We also assessed the effects saponins and related compounds on DPPG. The temperature of the main transition of untreated DPPG vesicles was 41.3 °C, with a half-width of the main peak of about 0.7 °C, and the enthalpy was about 10 kcal/mol (Figure 4c). The addition of tested compounds to the DPPG liposomes did not practically alter the *T_m_*, *T_1/2_*, and ∆∆*H* values (Figure 4c, Table 3, Table S2 Supplementary materials). Thus, all tested chemicals, including positively charged solasodine, did not demonstrate any effects on the membranes composed of negatively charged DPPG, in contrast to bilayers made from DPPC. Taking into account the difference in hydration between DPPC and DPPG membranes [29,30,31] this fact might also indicate the importance of the interactions of saponins and related compounds with the membrane water.

The lack of correlation between LogD_o/w_ and −Δ*T_m_*- or Δ*T_1/2_*-values (Table 1 and Table 2) demonstrates that the ability of tested compounds to alter lipid melting does not depend on the lipophilicity of their molecules.

### 2.5. The Effect of Saponins and Related Compounds on the Calcein Leakage

**Figure 5:**
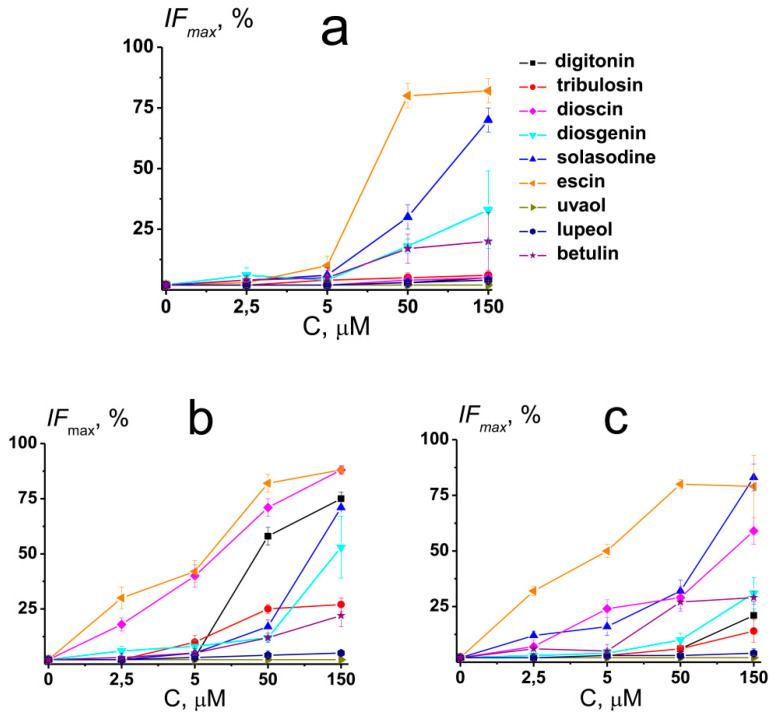
The dependence of relative fluorescence of calcein (*IF_max_*,%) leaked from POPC (**a**), POPC/CHOL (**b**), and POPG (**c**) vesicles on the concentration of different saponins and related compounds. The relationship between the color line and the compound is given on the figure.

To obtain further deep insights into the mechanisms of action of saponins and related compounds on membrane elastics, the release of the fluorescent marker calcein from unilamellar liposomes of different compositions after the introduction of the agents was studied. Figure 5 shows the dependence of maximal calcein leakage from large unilamellar vesicles made from POPC, POPC/CHOL (67/33 mol %), and POPG on compound concentration. The magnitude of the agent-induced maximal *IF_max_* is presented in Table S3 (Supplementary materials). 

The effects of tested compounds on membrane permeability for calcein at 5 µM were practically negligible, with the exception of dioscin and ecsin affecting POPC/CHOL and POPG-vesicles. At 50 µM, the efficacy of the tested agents to disengage the fluorescence marker from POPC vesicles decreased in the following order: escin (about 80%) > solasodine (about 30%) >> diosgenin ≈ betulin (about 20%) > digitonin ≈ tribulosin ≈ dioscin ≈ uvaol ≈ lupeol (less than 10 %) (Figure 5a and Table S3 Supplementary materials). In the case of POPC/CHOL liposomes, *IF_max_* decreased in the following order: escin (about 80%) > dioscin (about 70%) > digitonin (about 60%) > tribulosin ≈ solasodine (about 20%) > diosgenin ≈ betulin (about 10%) > uvaol ≈ lupeol (less than 10 %), and in the case of POPG liposomes, *IF_max_* decreased in the following order: escin (about 80%) > solasodine ≈ dioscin (about 30%) > betulin (about 20%) > diosgenin (about 10%) > digitonin ≈ tribulosin ≈ uvaol ≈ lupeol (less than 10 %) (Figure 5b,c and Table S3 Supplementary materials). At 150 µM, the efficiency row of agent ability to increase the permeability of POPC liposomes for calcein was not practically changed (Figure 5a and Table S3 Supplementary materials). At the same time, the efficacy of the tested compounds to disengage the fluorescence marker from POPC/CHOL and POPG vesicles at 150 µM was modified. In the case of POPC/CHOL liposomes, it decreased in the order: escin ≈ dioscin (about 90%) > digitonin ≈ solasodine (about 70%) > diosgenin (about 50%) > tribulosin (about 30%) > betulin (about 20%) > uvaol ≈ lupeol (less than 10 %) (Figure 5b and Table S3 Supplementary materials). In the case of POPG vesicles, *IF_max_* decreased in the following order: escin ≈ solasodine (about 80%) > dioscin (about 60%) > diosgenin (about 30%) > digitonin ≈ betulin (about 20%) > tribulosin (about 10%) > uvaol ≈ lupeol (less than 10 %), respectively (Figure 5c and Table S3 Supplementary materials).

One can notice that the effects of sapogenins (diosgenin, betulin, uvaol, and lupeol) and nonglycosylated alkaloid solasodine did not practically depend on the lipid composition of the vesicles. The ability of steroid saponins (digitonin, tribulosin, and dioscin) to disengage calcein from lipid vesicles decreased in the order of POPC/CHOL > POPG > POPC, while in the case of triterpenic saponin escin, it decreased in the order of POPC/CHOL ≈ POPG ≥ POPC. The ability of saponins and related compounds to disengage the fluorescent marker from lipid vesicles might be determined by their abilities to disorder membrane lipids and induce the positive curvature stress due to an unbalance between the polar and unpolar bilayer regions. The formation of the structures of the positive curvature, in particular, micellar-like, in the presence of tested agents can facilitate ion (Figure 2, Figure S1 Supplementary materials) and calcein leakage through the membranes. The ion-permeable defects induced by saponins and related compounds can serve as additional transport pathways for the fluorescence marker, and the more pronounced effect of tested compounds on the CHOL-enriched membranes might be explained by a more pronounced pore-forming ability of the chemicals in the bilayers composed of CHOL (Figure S1 Supplementary materials). The relationship between the induction of positive curvature stress, the formation of transient defects or pores, and an increase in membrane permeabilization by saponins has also been repeatedly emphasized in the review by Lorent et al. [6].

Figure S2 (Supplementary materials) shows the time dependence of relative fluorescence of calcein leaked from liposomes of different compositions in the presence of 50 µM of saponins and related compounds. The kinetics accelerated (time constant decreased) as the concentration of agent increased (Table S3 Supplementary materials). At lower diosgenin, solasodine, escin, and betulin concentrations (5–50 µM), the kinetics of the calcein release from POPC-vesicles was substantially slower than from POPC/CHOL and POPG liposomes, and the calcein leakage was characterized by similar kinetics independent of lipid composition of vesicles at 150 µM of the tested compounds. It also seems that sapogenins were characterized by slower kinetics compared to saponins. 

The discovered significance of sapogenin glycosylation for liposome permeabilization is in agreement with the results of Keukens et al. [12] and Nishikawa et al. [8]. Keukens et al. [12] found that intact tetra- and the tri-saccharide moieties of α-tomatine and α -chaconine, respectively, were of a key importance to those molecules to produce significant leakage of carboxyfluorescein from vesicles composed of equimolar mixture of phosphatidylcholine and CHOL: a deleting one or more mono-saccharides led to almost complete loss of ability of the alkaloid glycosides to increase membrane permeability for the fluorescent marker. Nishikawa et al. [8] showed that the ability to induce an increase in the permeability of CHOL-enriched liposomes decreases in the order: digitonin (containing the penta-saccharide moiety) ≈ desglucodigitonin (containing tetra-saccharide moiety) > glucosyl-galactosyl-digitogenin (containing bi-saccharide moiety) > galactosyl-digitogenin (containing mono-saccharide moiety and having no activity). Moreover, similar relative efficiency of digitonins was found in their induction of erythrocyte hemolysis demonstrating the coincidence of membrane perturbation mechanisms for lipid vesicles and red blood cells.

To distinguish between whether the leakage induced by the tested compounds is graded or “all-or-none”, we assessed the quenching factor (*Q*). In the case of graded process, it is assumed that calcein leaks out of all vesicles treated by agent, the gradient of fluorescence marker between inner content of liposomes and surrounding media decreases, and, after repassing through a column, modified vesicles indicate a smaller quenching factor. In the case of “all-or-none” mechanism, a portion of the liposomes is destroyed by the chemical with complete depletion of vesicle contents, while the remaining liposomes and their contents remain intact. Thus, in the case of “all-or-none” leakage, the quenching ratio of recovered vesicles should be unchanged compared to untreated liposomes [32,33]. After incubation of the POPC and POPC/CHOL liposomes with 50 µM of digitonin, diosgenin, uvaol, lupeol, and betulin for 1 h, and removal of the free calcein by gel filtration, *Q* decreased from 6–7 to 5–6, while in the presence of tribulosin, dioscin, solasodine, and escin, *Q* dropped to 3–4. Moreover, *Q* further decreased of about 1–2 units as the agent concentration increased up to 150 µM. These results indicate that the leakage of the marker under the action of all tested chemicals involves a graded process that cannot be attributed to full liposome destruction. At the same time, the results do not exclude the possibility of micelle formation and simultaneous destruction of a portion of the vesicles under the saponin action.

### 2.6. The Influence of Saponins and Related Compounds on the Mechanosensitive ion Channels Induced by Various Antifungals

Furthermore, to test the idea that saponins and related compounds can affect ion channels via alterations in the elastic properties of membranes, we used lipid pores formed by antifungal polyene macrolide antibiotic nystatin (NyS) and antifungal cyclic lipopeptide syringomycin E (SrE), which are known to be sensitive to changes in the membrane elastics induced by the adsorption of various membrane active agents [34,35,36]. Table 4 presents the mean ratios of the SrE and NyS produced steady-state transmembrane current in the presence and absence of dioscin and uvaol (*I_∞_/I_∞_^0^*).

Dioscin and uvaol at a concentration of 50 μM in the bathing solution did not affect the steady-state NyS-induced current through CHOL-enriched membranes. At the same time, the addition of dioscin led to an increase in the steady-state SrE-induced membrane conductance (*I_∞_/I_∞_^0^* was equal to 19.1 ± 6.8), while uvaol did not practically affect the SrE multichannel activity (*I_∞_/I_∞_^0^* was equal to 1.1 ± 0.1). The observed effect of dioscin on SrE-induced *I_∞_* is in agreement with its significant efficacy to alter DPPC melting. The absence of dioscin effect on NyS-pores might be related to its less pronounced effect on the thermotropic behavior of CHOL-enriched bilayers compared to CHOL-free membranes.

## 3. Materials and Methods

### 3.1. Chemical Reagents

**Figure 6:**
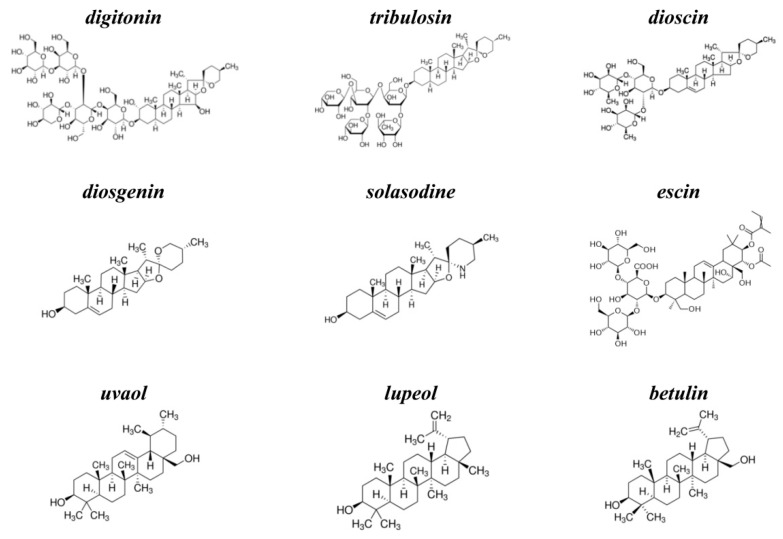
The chemical structures of tested agents: steroid saponins, digitonin, tribulosin, dioscin; steroid sapogenin, diosgenin; steroid alkaloid, solasodine; triterpenic saponin, escin; and triterpenic sapogenins, uvaol, lupeol, and betulin.

KCl, NaCl, HEPES, EDTA, nonactin, pentane, ethanol, methanol, calcein, triton X-100, sephadex G-50, digitonin, tribulosin, dioscin, diosgenin, solasodine, escin, uvaol, lupeol, betulin, nystatin (NyS), and gramicidin A (GrA) were purchased from Sigma-Aldrich (St. Louis, USA). Syringomycin E (SyrE) was isolated and purified as described previously [37], and kindly offered by Dr. J.Y. Takemoto (Utah State University, USA). The chemical structures of tested agents are presented in Figure 6. The purity of saponins and related compounds was ≥95% (except for betulin (≥98%), lupeol (≥94%), diosgenin (≥93%), and digitonin (~50%)). All experiments were performed at room temperature (25 °C).

Lipids, 1,2-diphytanoil-*sn*-glycero-3-phosphocholine (DPhPC), 1-O-hexadecyl-2-oleoyl-*sn*-glycero-3-phosphocholine (HOPC), 1-palmitoyl-2-oleoyl-*sn*-glycero-3-phosphocholine (POPC), 1-palmitoyl-2-oleoyl-*sn*-glycero-3-phospho-(1’-*rac*-glycerol) (POPG), 1,2-dipalmitoyl-*sn*-glycero-3-phosphocholine (DPPC), 1,2-dipalmitoyl-*sn*-glycero-3-phospho-(1’-*rac*-glycerol) (DPPG), and cholesterol (CHOL) were obtained from Avanti Polar Lipids (Pelham, NY, USA).

### 3.2. Electrophysiological Method for Measuring the Membrane Boundary Potential

Virtually solvent-free planar lipid bilayers were prepared using a monolayer-opposition technique [38] on a 50-µm diameter aperture in a 10-µm thick Teflon film separating the two (*cis*- and *trans*-) compartments of the Teflon chamber. The aperture was pretreated with hexadecane. Lipid bilayers were made from pure DPhPC (or HOPC) or mixtures of 67 mol% DPhPC and 33 mol% CHOL. The steady-state conductance of K^+^-nonactin was modulated via the two-sided addition of saponins and related compounds from mM stock solutions in water or ethanol to the membrane-bathing solution (0.1 M KCl, 5 mM HEPES, pH 7.4) to obtain a final concentration ranging from 2.5 to 150 μM. The conductance of the lipid bilayer was determined by measuring *I* at a constant transmembrane voltage (*V* = 50 mV). The subsequent calculations were performed assuming that the membrane conductance is related to the membrane boundary potential (φ*_b_*), the electric potential drop between the aqueous solution and the bilayer hydrophobic core, by the Boltzmann distribution [39]. The change in the membrane boundary potential at the adsorption of saponins and related compounds was averaged from 4 to 6 bilayers (mean ± SD, *p* ≤ 0.05).

### 3.3. Reconstitution of Ion Channels into Lipid Bilayers

Virtually solvent-free planar lipid bilayers were prepared using a monolayer-opposition technique [38], as described above. Lipid bilayers were made from DPhPC or a mixture of DPhPC/CHOL (67/33 mol%) and bathed in 0.1, 1.0, or 2.0 M KCl at pH 7.4. After the membrane was completely formed and stabilized, a stock solution of GrA, SrE, and NyS (in ethanol, water at pH 3.0, and DMSO) was added to both-side (GrA) or *cis*-side (SrE or NyS) of the chamber up to 0.1–0.3 μM, 2–10 μM, and 5–15 μM, respectively. Saponins and related compounds were added to both sides of the membrane up to 50 µM. 

The conductance of single GrA channels and pores induced by saponins and related compounds (*g*) was defined as the ratio between the current flowing through a single pore (*i*) and *V*. The GrA channel conductance fluctuation and dwell time (*τ*) histograms were made for the transmembrane voltage of 100 mV. The total number of events used for the channel amplitude and dwell time analysis was 500–1000 and 1500–2500, respectively. Peaks on the conductance and dwell time histograms were fitted by the normal density and exponential functions, respectively. The χ2 criterion was applied (*p* ≤ 0.05).

A steady-state transmembrane current induced by SrE or NyS (*I_∞_*) was used to assess the alteration in channel-forming activity of the antibiotics by two-sided addition of the saponins and related compounds. The mean ratios (*I_∞_*/*I_∞_^0^*) of the macroscopic currents after (*I_∞_*) and before (*I_∞_^0^*) two-sided addition of saponins and related compounds were averaged from 3 to 5 bilayers (mean ± sd).

Ag/AgCl electrodes with 1.5 % agarose/2 M KCl bridges were used to apply *V* and measure the transmembrane current. Here, "positive voltage" refers to the case in which the *cis*-side compartment is positive with respect to the *trans*-side. The current was measured using an Axopatch 200B amplifier (Molecular Devices, LLC, Orlean, CA, USA) in the voltage clamp mode. Data were digitized using a Digidata 1440A and analyzed using pClamp 10.0 (Molecular Devices, LLC, Orlean, CA, USA) and Origin 8.0 (OriginLab Corporation, Northampton, MA, USA). Data were acquired at a sampling frequency of 5 kHz using low-pass filtering at 200 Hz, and the current tracks were processed through an 8-pole Bessel 100-kHz filter.

### 3.4. Calcein Release Assay

Large unilamellar vesicles were prepared from pure POPC (or POPG) and a mixture of POPC/CHOL (67/33 mol%) by extrusion. Lipid stock in chloroform was dried under a gentle stream of nitrogen. Dry lipid film was hydrated by a buffer (35 mM calcein, 10 mM HEPES, pH 7.4). The suspension was subjected to five freeze-thaw cycles and then passed through a 100 nm Nuclepore polycarbonate membrane 13 times. The calcein that was not entrapped in the vesicles was removed by gel filtration with a Sephadex G-50 column to replace the buffer outside the liposomes with calcein-free solution (150 mM NaCl, 1 mM EDTA, 10 mM HEPES, pH 7.4). Calcein in vesicles fluoresces very poorly because of strong self-quenching at millimolar concentrations, while the fluorescence of disengaged calcein in the surrounding media correlates to the membrane stability and integrity in the absence and presence of saponins and related compounds.

The time-dependence of calcein fluorescence de-quenching induced by saponins and related compounds at a final concentration of 2.5, 5, 50, and 150 μM was measured at 10–30 min. The degree of calcein release was determined using a Fluorat-02-Panorama spectrofluorimeter (Lumex, Saint-Petersburg, Russia)**.** The excitation wavelength was 490 nm, and the emission wavelength was 520 nm. Triton X-100 was added to a final concentration of 10 mM to each sample in order to completely disrupt the liposomes, and the intensity after releasing the total amount of calcein from the liposomes was measured. 

To describe the dependence of permeabilization of the liposomal membranes on the concentration of saponins and related compounds and time, the relative intensity of calcein fluorescence (*IF*, %) was used:

(1) $$IF=\frac{I-I_{0}}{I_{\max}/0.9-I_{0}}\cdot 100\%$$

where *I* and *I*_0_ were the calcein fluorescence intensity in the sample in the presence and absence of saponins and related compounds, respectively, and *I_max_* was the maximal fluorescence of the sample after lysis of liposomes by triton X-100. A factor of 0.9 was introduced to account for the dilution of the sample by triton X-100. The time dependences of calcein leakage were fitted with one-exponential decay functions to assess the time constants (*t*). The maximal leakage of fluorescent marker from vesicles (*IF_max_*) was determined after the calcein release had reached a steady-state value.

To distinguish between whether the leakage induced by saponins and related compounds is graded or “all-or-none”, we assessed the quenching factor (*Q*). In the case of “all-or-none” leakage, the recovered vesicles should exhibit the same quenching ratio as the untreated vesicles, whereas in the case of graded release, the quenching ratio should be reduced [32]. After incubation of the POPC, POPC/CHOL, and POPG liposomes with 50 or 150 µM of saponins and related compounds for 30 min and removal of the free calcein and micellized lipids by gel filtration, *Q* was measured. 

The characteristic parameters of calcein release from liposomes in the presence of tested compounds were averaged from 2–5 experiments for each system (mean ± sd, *p* ≤ 0.05).

### 3.5. Differential Scanning Microcalorimetry of Liposomal Suspensions

Differential scanning microcalorimetry experiments were performed by a μDSC 7EVO microcalorimeter (Setaram, France). Giant unilamellar vesicles were prepared from pure DPPC (or DPPG) or a mixture of DPPC/CHOL (90/10 mol%) with Nanion Vesicle Prep Pro setup (Nanion Technologies, USA) by electroformation (standard protocol, 3 V, 10 Hz, 1 h, 55 °C). The resulting liposome suspension contained 3 mM lipid and was buffered by 5 mM HEPES at pH 7.4. Saponins and related compounds were added to aliquots to obtain a final concentration of 50 μM. The liposomal suspension was heated at a constant rate of 0.2 °C·min^−1^. The reversibility of the thermal transitions was assessed by reheating the sample immediately after the cooling step from the previous scan. The temperature dependence of the excess heat capacity was analyzed using Calisto Processing (Setaram, France). The lipid thermograms were characterized by the presence of two peaks referring to less energetic pre-transition and more energetic main phase transition. The main peak of pure DPPC, DPPG, or mixture of DPPC and CHOL (90/10 mol%) in the absence and presence of tested compounds was described by the maximum temperature (*T_m_*), the half-width (*T_1/2_*), characterizing the cooperativity of the melting process, and the changes in the enthalpy (Δ*H*). The ∆*T_m_,* ∆*T_1/2_* and ∆∆*H* values were averaged from 2–3 independent experiments for each lipid/chemical system (mean ± sd, *p* ≤ 0.05).

### 3.6. Correlation Analysis between the Altered Membrane Parameter and Octanol/Water Partition Coefficient of Tested Compounds

The logarithms of the octanol/water distribution coefficients of tested compounds at pH 7.4 (LogD_o/w_) were predicted by ChemAxon. Pearson’s correlation coefficient was applied to estimate the relationships between agent lipophilicity expressed in units of (LogD_o/w_) and chemical-induced changes in the physical properties of lipid bilayers (−Δφ*_b_*(max), *K*, *IF_max_*, −Δ*T_m_*, Δ*T_1/2_*) using Excel. To determine the confidence interval of the sample correlation coefficient, the Fisher transformation at a confidence level of 0.1 was used.

## 4. Conclusions

1. The ability of saponins and related compounds to induce transmembrane pores depends on the agent type and membrane lipid composition. Cholesterol enhances the pore-forming and detergent-like activity of the compounds. All tested agents of steroid nature (digitonin, tribulosin, dioscin, diosgenin, and solasodine), and only one triterpenic sapogenin (betulin) among four studied triterpens are characterized by the ability to form pores in CHOL-enriched bilayers.
2. Saponins (digitonin, tribulosin, dioscin, and escin) might decrease the membrane boundary potential. Sapogenins (diosgenin, uvaol, lupeol, and betulin) and nonglycosylated alkaloid solasodine are not characterized by this ability. Moreover, saponins can regulate the fluxes through ion channels, in particular, through cation-selective gramicidin A pores, by changing the transmembrane distribution of the electric potential. The dependence of the dipole-modifying ability on sapogenin glycosylation, the lack of the saponin effects on plasmanylcholine membranes as well as the absence of the correlation between the changes in the dipole potential and the lipophilicity of the compounds, indicate that saponins affect the dipole potential by altering the membrane hydration layer.
3. The ability of saponins and related compounds to affect the phase behavior of membrane-forming lipids depends on the structure of lipophilic part of molecule (steroid or triterpenic). Steroids (digitonin, tribulosin, dioscin, diosgenin, and solasodine) are characterized by more pronounced effects in CHOL-free DPPC bilayers than in CHOL-enriched DPPC membranes. Triterpenes (uvaol, lupeol, and betulin) show an inverse relationship. Triterpenic saponin escin has no effect on lipid melting independent of the vesicle composition. The dependence of the effects on the structure of the lipophilic core of molecule and the presence of CHOL in the bilayer might indicate that tested agents affect lipid melting mainly through immersion into membrane and disordering of the lipids. The influence of saponins on the elastic properties of the membrane determines the possibility of regulating mechanosensitive ion channels, such as asymmetric peptide-lipid pores formed by syringomycin E.
4. The effects of saponins and related compounds on the membrane permeability for the fluorescent marker dramatically depend on the agent structure and the liposome composition. The sapogenins (diosgenin, uvaol, lupeol, and betulin) and nonglycosylated alkaloid solasodine, increase permeability of POPC, POPC/Chol, and POPG vesicles for calcein in the same manner. Steroid saponins (digitonin, tribulosin, and dioscin) are characterized by the greatest efficiency in POPC/CHOL bilayers, while triterpenic saponin escin is almost equally effective in POPC/Chol and POPG membranes. The ability of saponins and related compounds to disengage the fluorescent marker from lipid vesicles might be determined by their abilities to disorder membrane lipids and induce the positive membrane curvature stress. The formation of the micellar-like structures in the presence of the tested chemicals might also facilitate ion and calcein transport through the membranes.

## Figures and Tables

**Table 1:** The characteristic parameters of the action of the tested compounds on the electrical properties of the model lipid membranes.

Compound	Charge *	LogD_o/w_ *	*C*, μM ^&^		−Δ*φ_b_*(max), mV		
			DPhPC	DPhPC/CHOL	DPhPC	DPhPC/CHOL	HOPC
*digitonin*	0.00	−4.96	150 ± 25	75 ± 25	36 ± 4	27 ± 5	4 ± 2
*tribulosin*	*na*	*na*	200 ± 25	125 ± 25	47 ± 6	22 ± 9	6 ± 2
*dioscin*	0.00	1.71	150 ± 25	100 ± 25	39 ± 8	26 ± 9	5 ± 1
*diosgenin*	0.00	4.93	150 ± 25	125 ± 25	6 ± 2	5 ± 3	3 ± 2
*solasodine*	0.99	2.50	150 ± 25	100 ± 25	5 ± 2	7 ± 2	2 ± 1
*escin*	−1.00	−4.29	150 ± 25	75 ± 25	20 ± 5	16 ± 4	5 ± 2
*uvaol*	0.00	6.11	200 ± 25	150 ± 25	1 ± 1	1 ± 1	5 ± 3
*lupeol*	0.00	7.45	200 ± 25	150 ± 25	1 ± 1	1 ± 1	3 ± 1
*betulin*	0.00	6.17	150 ± 25	100 ± 25	1 ± 1	3 ± 1	3 ± 2

* the values of the total electrical charge and LogD_o/w_ (the logarithm of octanol/water distribution coefficient at pH 7.4) were predicted by ChemAxon. &—the threshold concentrations of saponins and related compounds in the membrane bathing solutions (0.1 M KCl pH 7.4) required breaking the bilayer. Δ*φ_b_*(max)—the maximum change in the membrane boundary potential.

**Table 2:** The parameters of single GrA pores in the absence and presence of 50 μM of tribulosin and lupeol: *G_SC_*—the pore conductance at *V* = 100 mV; τ—the dwell time of GrA channel. The lipid bilayers were composed of DPhPC and bathed in 1 M KCl (pH 7.4).

Compound	*G_SC_*, pS	τ, s
*–*	21.5 ± 0.5	3.0 ± 1.0	
*tribulosin*	23.6 ± 0.8	4.7 ± 0.3	
*lupeol*	20.9 ± 0.6	3.1 ± 0.6	

**Table 3:** Parameters charactering the effects of saponins and related compounds on the elastic properties of lipid bilayers at 50 μM: Δ*T_m_*—the changes in the main transition temperature; Δ*T_1/2_*—the changes in the half-width of the main peak.

Compound	−∆*T_m_*, °C			∆*T_1/2_*, °C		
	DPPC	DPPC/CHOL	DPPG	DPPC	DPPC/CHOL	DPPG
*digitonin*	0.7 ± 0.1	0.4 ± 0.1	0	0.4 ± 0.1	0.1 ± 0.1	0.1 ± 0.1
*tribulosin*	0.9 ± 0.2	0.5 ± 0.1	0	0.3 ± 0.1	0.1 ± 0.1	0.1 ± 0.1
*dioscin*	0.8 ± 0.2	0.5 ± 0.1	0.1 ± 0.1	0.7 ± 0.2	0.5 ± 0.2	0.2 ± 0.1
*diosgenin*	0.4 ± 0.1	0.2 ± 0.1	0	0.5 ± 0.1	0.1 ± 0.1	0.1 ± 0.1
*solasodine*	0.5 ± 0.1	0.3 ± 0.1	0	0.4 ± 0.1	0.2 ± 0.1	0.1 ± 0.1
*escin*	0	0	0	0	0	0
*uvaol*	0.2 ± 0.1	0.8 ± 0.1	0.1 ± 0.1	0.1 ± 0.1	0.3 ± 0.1	0.1 ± 0.1
*lupeol*	0.3 ± 0.1	0.4 ± 0.1	0.1 ± 0.1	0.1 ± 0.1	0.1 ± 0.1	0.2 ± 0.1
*betulin*	0.2 ± 0.1	0.5 ± 0.1	0.1 ± 0.1	0.1 ± 0.1	0.1 ± 0.1	0.1 ± 0.1

The temperatures of the main transition of untreated DPPC (DPPG) are approximately 41.2 (41.0) °C, respectively, with the half-width of the main peak of approximately 0.6 (0.7) °C. These data are in a good agreement with the results of [28,29].

**Table 4:** The ratio of the transmembrane current induced by the antimycotics in the presence and absence of 50 μM of dioscin and uvaol. The SrE modified lipid bilayers were composed of DPhPC and bathed in 0.1 M KCl (pH 7.4) and NyS-altered bilayers were formed from DPhPC/CHOL (67/33 mol%) and bathed in 2 M KCl (pH 7.4).

Compound	SrE	NyS
*dioscin*	19.1 ± 6.8	1.0 ± 0.1
*uvaol*	1.1 ± 0.1	0.8 ± 0.1

## Supplementary Materials

The following are available online at https://www.mdpi.com/1422-0067/22/6/3167/s1, Figure S1: Current fluctuations corresponding to the openings and closures of single ion-permeable pores induced by saponins and related compounds in cholesterol-containing lipid bilayers, Figure S2: The time dependence of relative fluorescence of calcein leaked from lipid vesicles in the presence of saponins and related compounds, Table S1: The desorption constants of tested compounds; Table S2: The changes in the enthalpy of the main phase transition of different lipids at 50 μM of saponins and related compounds; Table S3: The characteristics of action of saponins and related compounds on the calcein release from liposomes of different composition.
