# Supplementary material for: Is the Membrane Lipid Matrix a Key Target for Action of Pharmacologically Active Plant Saponins?
Source: Int J Mol Sci. 2021 Mar 20;22(6):3167. doi: 10.3390/ijms22063167 (PMC8003763; doi:10.3390/ijms22063167)
Supplement: Supplementary file 1 [file ijms-22-03167-s001.pdf]

Supplementary material

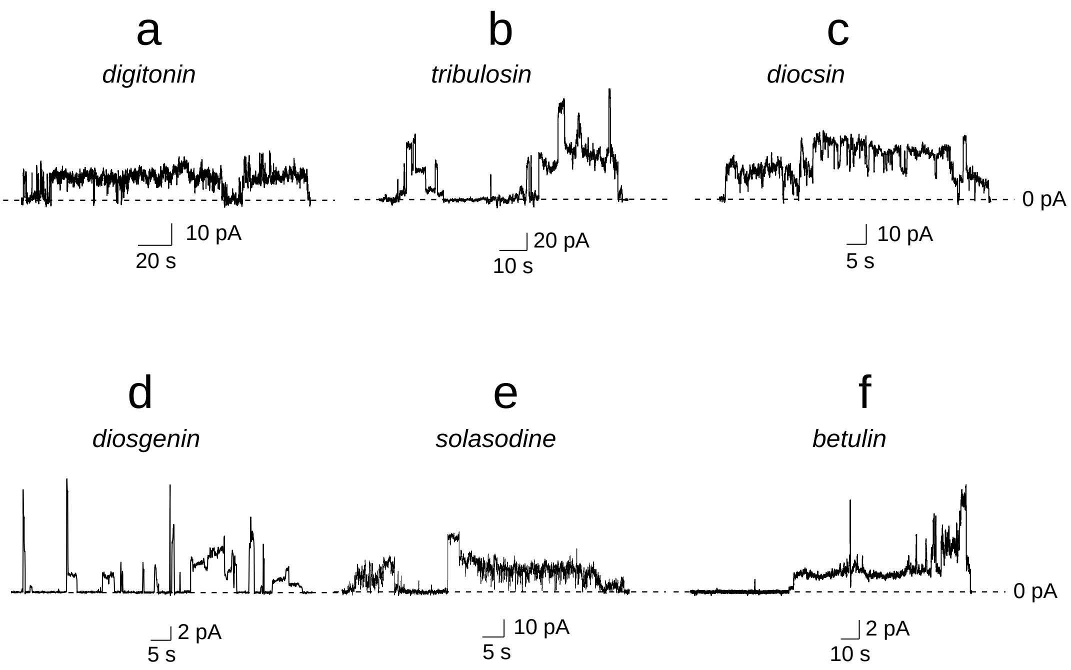

Figure S1. Current fluctuations corresponding to the openings and closures of single ion-permeable pores induced by 75 μM of digitonin (**a**), 125 μM of tribulosin (**b**), 100 μM of dioscin (**c**), 125 μM of diosgenin (**d**), 100 μM of solasodine (**e**), and 100 μM of betulin (**f**) in lipid bilayers composed of DPhPC/CHOL and bathed in 0.1 M KCl (pH 7.4). *V* was equal to 100 mV.

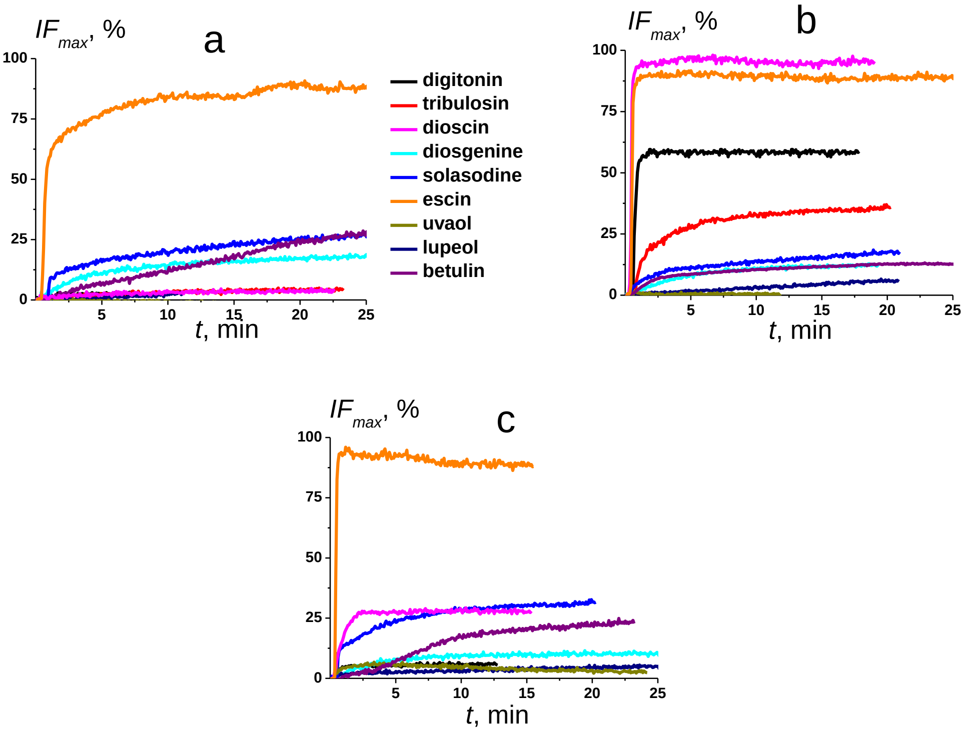

Figure S2. The time dependence of relative fluorescence of calcein (IF_{max},%) leaked from POPC (**a**), POPC/CHOL (**b**), and POPG (**c**) vesicles. 50 μM of saponins or related compounds were added into the

liposomal suspension at the first time point. The relationship between the color line and the compound is given in the figure.

Table S1. The desorption constants of the tested compounds (*K*, μM)

compound	*DPhPC*	*DPhPC/CHOL*
digitonin	14 ± 2	11 ± 3
tribulosin	17 ± 4	12 ± 3
dioscin	9 ± 1	4 ± 2
diosgenin	–#	–#
solasodine	–#	–#
escin	6 ± 2	3 ± 2
uvaol	–#	–#
lupeol	–#	–#
betulin	–#	–#

desorption constant cannot be determined due to the small absolute value of $\Delta\phi_b$(max).

Table S2. The changes in the enthalpy of the main phase transition of different lipids at 50 μM of saponins and related compounds (*-ΔΔH, kcal/mol*)

compound	*DPPC*	*DPPC/CHOL*	*DPPG*
digitonin	0.5 ± 0.1	0.6 ± 0.2	0.6 ± 0.3
tribulosin	0.1 ± 0.1	0.7 ± 0.2	0.4 ± 0.2
dioscin	0.3 ± 0.2	0.5 ± 0.1	0.4 ± 0.2
diosgenin	0.6 ± 0.2	0.9 ± 0.2	0.5 ± 0.3
solasodine	0.3 ± 0.2	0.5 ± 0.4	0.1 ± 0.1
escin	0.1 ± 0.1	0.5 ± 0.2	0.2 ± 0.1
uvaol	0.1 ± 0.1	1.0 ± 0.4	0.1 ± 0.1
lupeol	0.1 ± 0.1	1.5 ± 0.3	0.3 ± 0.1
betulin	0.2 ± 0.1	1.4 ± 0.3	0.4 ± 0.2

The enthalpy of the main phase transition of untreated DPPC, DPPC/CHOL, and DPPG is 9.0, 9.5, and 10 kcal/mol respectively.

Table S3. The characteristics of action of saponins and related compounds on the calcein release from liposomes of different composition: IF_{max} – the maximal leakage of fluorescent marker from vesicles; *t* – characteristic parameter of the time dependences of marker leakage.

	C, μM	IF_{max}*, %*			*t, min*		
compound		*POPC*	*POPC/CHOL*	*POPG*	*POPC*	*POPC/CHOL*	*POPG*
digitonin	5	2 ± 2	3 ± 2	3 ± 2	–	–	–
	50	3 ± 2	58 ± 4	6 ± 2	–	0.3 ± 0.1	–
	150	5 ± 2	74 ± 3	21 ± 6	–	0.1 ± 0.1	0.1 ± 0.1
tribulosin	5	4 ± 2	9 ± 3	3 ± 2	–	–	–
	50	5 ± 2	25 ± 2	6 ± 2	–	1.8 ± 0.5	–
	150	6 ± 3	27 ± 3	14 ± 5	–	0.6 ± 0.2	1.3 ± 0.1
dioscin	5	2 ± 2	40 ± 5	24 ± 4	–	0.5 ± 0.1	3.9 ± 0.4
	50	4 ± 2	71 ± 4	29 ± 5	–	0.2 ± 0.1	0.7 ± 0.2
	150	5 ± 3	88 ± 2	59 ± 6	–	0.1 ± 0.1	0.5 ± 0.2

diosgenin	5	6 ± 4	8 ± 3	4 ± 2	–	–	–
	50	18 ± 3	12 ± 2	10 ± 3	6.7 ± 0.8	4.3 ± 0.7	–
	150	33 ± 8	53 ± 14	31 ± 7	5.8 ± 1.3	4.8 ± 1.5	7 ± 1
solasodine	5	6 ± 3	5 ± 2	16 ± 4	–	–	0.9 ± 0.1
	50	30 ± 5	17 ± 3	32 ± 5	11.7 ± 0.5	1.8 ± 0.4	3.8 ± 0.5
	150	70 ± 5	71 ± 3	83 ± 6	1.8 ± 0.2	1.5 ± 0.9	2.3 ± 0.2
escin	5	10 ± 4	42 ± 5	50 ± 3	8.4 ± 0.5	1.2 ± 0.4	0.4 ± 0.1
	50	80 ± 5	82 ± 4	79 ± 2	1.3 ± 0.1	0.2 ± 0.1	0.1 ± 0.1
	150	82 ± 5	88 ± 2	80 ± 5	0.2 ± 0.1	0.1 ± 0.1	0.1 ± 0.1
uvaol	5	2 ± 1	2 ± 1	2 ± 2	–	–	–
	50	2 ± 2	2 ± 2	2 ± 2	–	–	–
	150	2 ± 1	2 ± 1	2 ± 1	–	–	–
lupeol	5	2 ± 2	3 ± 2	3 ± 2	–	–	–
	50	3 ± 2	4 ± 2	3 ± 2	–	–	–
	150	4 ± 2	5 ± 1	4 ± 1	–	–	–
betulin	5	5 ± 3	5 ± 2	5 ± 3	–	–	–
	50	17 ± 6	14 ± 4	20 ± 4	56 ± 4	5 ± 1	11± 1
	150	20 ± 12	22 ± 3	22 ± 3	1.6 ± 0.3	3 ± 1	2 ± 1
